# 

**Published:** 1871-08

**Authors:** 


VOL. I. AUGUST 1871. NO. 8.

### THE GEORGIA MEDICAL COMPANION,

DEVOTED TO THE

### SCIENCE OF MEDICINE AND SURGERY.

EDITORS:

T. S. POWELL, M. D., and W. T. GOLDSMITH, M. D.

ASSOCIATE EDITORS:

ROBERT BATTEY, M. D., Rome, Ga.; R. C. WORD, M. D., Tunnel Hill, Ga.; JAMES A. LONG, M. D., LaGrange, Ga.; E. F. KNOTT, M. D., Griffin, Ga.; W. L. M. HARRIS, M. D., Greensboro, Ga.; W. C. MUSGROVE, M. D., Midville, Ga.; C. H. BASS, M. D., Lunatic Asylum, Ga.; JOHN C. DRAKE, M. D., Thomaston, Ga.; A. H. BRANTLY, M. D., Decatur, Ga.; J. J. KNOTT, M. D., Atlanta, Ga.; THOMAS BURDELL, M. D., Waynesboro Ga.; E. H. W. HUNTER, M. D., Louisville, Ga.; W. L. KIRKPATRICK, M. D., Cartersville, Ga.; V. G. HITT, M. D., Wooten's Station, Ga.; L. B. BURCHELLE, M. D., Midville, Ga.; HENRY H. BATTLE, M. D., Bethany, Ga.; W. B. HARVEY, M. D., Canton, Miss.; C. B. GALLOWAY, M. D., CANTON, Miss.; S. V. D. HILL, M. D., Macon, Miss.; EDWARD LEA, M. D., Holly Spring, Miss.; J. W. SHATTUCK, M. D., Columbus, Miss.; THOMAS P. LOCKWOOD, M. D., Crystal Spring, Miss.

*"QUICQUID PRÆCIPIES ESTO BREVIS."*

ATLANTA, GA.
FRANKLIN STEAM PRINTING HOUSE.
J. J. TOON, PROPRIETOR.

TERMS, - - - - $2 A YEAR, IN ADVANCE.

